# Visualisation of HER2 homodimers in single cells from HER2 overexpressing primary formalin fixed paraffin embedded tumour tissue

**DOI:** 10.1186/s10020-019-0108-z

**Published:** 2019-08-28

**Authors:** Diana B. Peckys, Daniela Hirsch, Timo Gaiser, Niels de Jonge

**Affiliations:** 1 Department of Biophysics, Saarland University, Homburg, Germany; 2 Institute for Pathology, University Medical Center Mannheim, Ruprecht-Karls University of Heidelberg, Mannheim, Germany; 3 INM – Leibniz Institute for New Materials, Campus D2-2, 66123 Saarbrücken, Germany; 4 Department of Physics, Saarland University, Saarbrücken, Germany

**Keywords:** HER2 homodimer, Dissociated cells, Biopsy sample, Scanning transmission electron microscopy, STEM, Graphene, Pair correlation function

## Abstract

**Background:**

HER2 is considered as one of the most important, predictive biomarkers in oncology. The diagnosis of HER2 positive cancer types such as breast- and gastric cancer is usually based on immunohistochemical HER2 staining of tumour tissue. However, the current immunohistochemical methods do not provide localized information about HER2’s functional state. In order to generate signals leading to cell growth and proliferation, the receptor spontaneously forms homodimers, a process that can differ between individual cancer cells.

**Materials and methods:**

HER2 overexpressing tumour cells were dissociated from formalin-fixed paraffin-embedded (FFPE) patient’s biopsy sections, subjected to a heat-induced antigen retrieval procedure, and immobilized on microchips. HER2 was specifically labelled via a two-step protocol involving the incubation with an Affibody-biotin compound followed by the binding of a streptavidin coated quantum dot (QD) nanoparticle. Cells with membrane bound HER2 were identified using fluorescence microscopy, coated with graphene to preserve their hydrated state, and subsequently examined by scanning transmission electron microscopy (STEM) to obtain the locations at the single molecule level. Label position data was statistically analysed via the pair correlation function, yielding information about the presence of HER2 homodimers.

**Results:**

Tumour cells from two biopsies, scored HER2 3+, and a HER2 negative control sample were examined. The specific labelling protocol was first tested for a sectioned tissue sample of HER2-overexpressing tumour. Subsequently, a protocol was optimized to study HER2 homodimerization in single cells dissociated from the tissue section. Electron microscopy data showed membrane bound HER2 in average densities of 201–689 proteins/μm^2^. An automated, statistical analysis of well over 200,000 of measured protein positions revealed the presence of HER2 homodimers in 33 and 55% of the analysed images for patient 1 and 2, respectively.

**Conclusions:**

We introduced an electron microscopy method capable of measuring the positions of individually labelled HER2 proteins in patient tumour cells from which information about the functional status of the receptor was derived. This method could take HER2 testing a step further by examining HER2 homodimerization directly out of tumour tissue and may become important for adjusting a personalized antibody-based drug therapy.

**Electronic supplementary material:**

The online version of this article (10.1186/s10020-019-0108-z) contains supplementary material, which is available to authorized users.

## Background

HER2 overexpression plays a pivotal role in breast and gastric cancer, as well as in many other types of cancer (Beuzeboc et al. 2001; Menard et al. 2001). It belongs to the growth factor receptor family, a subgroup of the tyrosine kinases, proteins that are key regulators of cellular proliferation, growth, and survival. Overexpression and/or mutational constitutive activation of these receptors cause aberrant signalling and proliferation. In order to start the intracellular growth-signalling cascade all members of the growth factor family need to interact by forming hetero- or homodimers (Hudziak et al. 1987; Yarden and Sliwkowski 2001). The outstanding role of HER2 within this family is that it can form signalling active dimers without the need for any ligand binding (Lonardo et al. 1990). Homodimerization occurs spontaneously when HER2 is overexpressed, which inevitably leads to increased cell proliferation and invasiveness (Hudziak et al. 1987; Di Fiore et al. 1987). HER2 is considered as one of the most important, predictive biomarkers in oncology (Allred et al. 2012). HER2 belongs to the exclusive group of oncogenic proteins for which a targeted therapy in the form of antibody-based drugs like Trastuzumab, Pertuzumab or T-DM1 are available. Given the large therapeutic benefit of these biological drugs for the individual patient, but also in view of the urging problem of drug resistance, an accurate determination of the patients' HER2 status, including information about the presence of signalling active homodimers, is crucial.

Here, we introduce a microscopy method capable of measuring the positions of individually labelled HER2 proteins in patient tumour cells. The spatial information was used to detect the presence of HER2 homodimers, and to examine other features of HER2’s spatial distribution in the plasma membrane such as its surface density. We first tested the specific HER2 labelling (Peckys et al. 2015) in standard tissue sections via fluorescence microscopy. Tumour regions were macro-dissected from 50 um-thick sections, dissociated into single cells, again examined using light microscopy, and finally prepared for electron microscopic examination. In order to preserve the plasma membrane, whole cells were prepared in hydrated state by covering them with graphene (Dahmke et al. 2017) protecting evaporation in the vacuum of the electron microscope. Cells of two patients were finally examined using STEM.

## Materials and methods

### Materials

EnVision detection system, peroxidase/diaminobenzidine, rabbit/mouse, cat # K5007, citrate, cat # S2369, and HER2 antibody, cat # A0485, for standard (Immunohistochemistry) IHC on tumour sections were obtained from Dako/Agilent*.* SuperFrost Plus microscope slides were from Daigger Scientific, Vernon Hills, IL, USA. Epitope retrieval solution (pH 6) was from Leica Biosystems, Wetzlar, Germany. Biotin conjugated Anti-HER2 Affibody was (ZHER2:477)2, from Affibody AB, Bromma, Sweden. Dispase, normal goat serum, QD (Qdot® 655 nm) streptavidin conjugate were from Thermo Fisher Scientific GmbH, Dreieich, Germany. ROTISOLV® high pressure liquid chromatography (HPLC) grade deionized water, acetone and ethanol, phosphate buffered saline (PBS) 10 × solution, electron microscopy grade glutaraldehyde (GA) 25% solution, D-saccharose, sodium chloride, glycine, biotin free and molecular biology grade bovine serum albumin fraction V (BSA), and sodium cacodylate trihydrate were from Carl Roth GmbH + Co. KG, Karlsruhe, Germany. Electron microscopy grade formaldehyde 16% solution was from Science Services GmbH, Munich, Germany. Histochoice Clearing Agent, collagenase IA, Tween, 0.01% poly-L-lysine (PLL) solution (mol wt 70,000-150,000), MAPTrix™ Reagent high MW, 0.5 mg/mL protein, sodium bicarbonate, sodium hydroxide, sodium tetraborate, sodium azide and boric acid were from Sigma-Aldrich Chemie GmbH, Munich, Germany. CELLVIEW cell culture dishes (35 mm) with 4 compartments and glass bottom were from Greiner Bio-One GmbH, Frickenhausen, Germany. Custom designed silicon microchips were purchased from DENSsolutions, Delft, Netherlands. The microchips had outer dimensions of 2.0 × 2.6 × 0.4 mm and each contained a central silicon nitride (SiN) membrane window with dimensions of 150 × 400 μm and a thickness of 50 nm. Trivial transfer multilayer graphene was purchased from, ACS Material LLC, Pasadena, CA, USA. NaCl_2_ crystals were from Plano GmbH, Wetzlar, Germany. All solutions, except those of HPLC grade, were filter-sterilized prior to use. If not indicated otherwise, all procedures were performed at room temperature (RT).

### Graphene preparation

Poly-methyl-methacrylate (PMMA) covered multi-layer (3 to 5 layers) graphene was used. To remove the PMMA layer and detach the graphene from the polymer substrate, the composite was submerged into a NaCl_2_ saturated, deionized water solution at 45° angle and the floating graphene-PMMA stack was then scooped up with a NaCl_2_ crystal (Dahmke et al. 2017; Weatherup et al. 2016). After baking it in an oven at 100 °C for 20 min, the stack on salt crystal was immersed into acetone for 30 min to remove the PMMA, and was subsequently air-dried. After this point, the graphene on salt was cut with a razor blade into into pieces of ~ 2 × 2 mm as needed to cover the samples.

### Preparation of SiN membrane microchips and cell culture dishes

To remove the protective resist layer from the silicon microchips they were incubated for 2-min in HPLC grade acetone, then rinsed for 2 min in HPLC grade ethanol, air-dried, and exposed for 5 min to plasma cleaning (ambient air plasma). Immediately afterwards, the microchips were incubated in 0.01% PLL for 5 min, rinsed twice with HPLC grade water, and incubated for 60 min, in 250 μl MAPTrix coating solution, corresponding to ~ 6 μg MAPTrix/cm^2^. MAPTrix contains components of the mussel adhesive protein, which, by absorption to the PLL coated surfaces turns them sticky, in order to robustly immobilize single, dispersed tumour cells. The MAPTrix 0.5 mg/mL stock solution was therefore diluted 1:8 in 0.1 M sodium bicarbonate buffer, pH 8.0, and the pH was raised by adding 1 M NaOH in a 1:50 ratio, to induce the absorption process, which was allowed to proceed for 1 h. Afterwards, and directly prior to the seeding of dissociated cells, the coated microchips were rinsed twice with HPLC grade water. Preparation of the glass bottom, four-compartment cell culture dishes was principally the same as for the microchips.

### HER2 immunohistochemistry on FFPE tissue sections

Tissue sections from three cases were prepared of which two were HER2 positive and one was HER2 negative. HER2 positivity was defined by immunohistochemistry and scoring was done in accordance with the current HER2 scoring guidelines (Wolff et al. 2018). The two positive cases were diagnostically assigned with HER2 score 3+ while the negative case did not show any relevant staining (HER2 score 0). The tissue sections were first deparaffinized in xylene (3 × 5 min). The slides were then rehydrated in a decreasing ethanol series (100, 100, 96, 80%, 2 min each) before rinsing in distilled water twice. Heat-induced antigen retrieval was performed by incubation in Epitope Retrieval Solution (pH 6), citrate in a water bath at 95 °C for 40 min. Primary HER2 antibody (1:500 dilution) was added to the tissue section for 30 min. Detection was done using the EnVision detection system, peroxidase/diaminobenzidin, rabbit/mouse, according to the manufacturer’s instructions.

### Preparation of single cell suspensions from FFPE tumour tissues

Single cell suspensions were prepared from 50 μm-thick tumour sections following the protocol described by Bolognesi et al. with some modifications (Bolognesi et al. 2016). Briefly, tumour samples were deparaffinized with Histochoice Clearing Agent at 65 °C followed by rehydration in an ethanol series (100, 70, 50%, 2 × 5 min each). Heat-induced antigen retrieval was performed by incubation in epitope retrieval solution (pH 6) at 80 °C for 60 min. After washing in PBS, tissue samples were enzymatically digested in 0.1% collagenase IA and 0.1% dispase at 37 °C for 30 min followed by mechanical dissociation with a syringe. Disintegrated cells were washed with PBS twice and then resuspended in PBS supplemented with 1% BSA and 0.05% Tween.

### Processing of dissociated tumour cells for HER2 labelling

Biotin conjugated Anti-HER2 Affibody (HER2-AFF-B) stock solution (20 μM) was adjusted to a final concentration of 200 nM in PBS supplemented with 1% normal goat serum (GS), and 1% BSA (GS-BSA-PBS). Streptavidin-QD655 stock solution (1 μM) was diluted 1:2 in 40 mM borate buffer (sodium tetraborate, boric acid, pH 8.3), and then diluted to a final concentration of 20 nM by adding PBS supplemented with 1% BSA (BSA-PBS). The dissociated cell suspensions were processed in batches of 500 or 250 μl volumes, handled in 1.5 ml microcentrifuge tubes, and using centrifugation forces of 2500 g and centrifugation times of 1 min per 100 μl of cell suspension. To diminish unspecific HER2-AFF-B binding the cells were washed once, and then incubated for 10 min, at 37 °C, in GS-BSA-PBS. The cell pellets were resuspended in 250 μl HER2-AFF-B labelling solution, and incubated for 10 min, at 37 °C. After 3 washing cycles with PBS, the cell pellets were resuspended in ~ 75 μl PBS. The water-rinsed, MAPTrix-coated microchips were placed in new wells of a 96-well plate and the concentrated cell suspensions were pipetted onto the wet microchips. During the following 1 h incubation time the cells sunk onto the microchip and firmly adhered. The microchip samples were then rinsed once with 0.1 M cacodylate buffer (CB) supplemented with 0.1 M saccharose (S), pH 7.4 (CB-S), followed by fixation with 3% FA in CB for 10 min. After another rinse with CB-S, 3 rinses with PBS, a 2-min incubation in 0.1 M glycine (GLY) in PBS, pH 7.4 (GLY-PBS), and a rinse with PBS, the samples were placed in 75 μl strep-QD labelling solution, and incubated for 12 min. This two-step labelling protocol ensured HER2 labelling in a 1:1 stoichiometry (Peckys et al. 2015). After rinsing 3 × with BSA-PBS the samples were imaged with light microscopy (LM). To ensure a stable chemical fixation of the biological material for EM imaging, the cells were fixed with 2% GA in CB-S, similar to the FA fixation described above. The samples were stored in BSA-PBS, supplemented with 0.02% SA, at 4 °C, until electron microscopy was performed, usually within the next few days.

### Light microscopy

Labelled- and formaldehyde-fixed cells on microchips were imaged in BSA-PBS using an inverted light microscope (DMI6000B, Leica, Germany). For this purpose, the microchips were placed upside-down in a glass bottom dish. Direct interference contrast (DIC) images were acquired from each sample yielding information about membrane topography, and fluorescence images were acquired for the detection of HER2-bound strep-QDs, using a filter cube “A” with a 340–380 nm excitation and a > 420 nm emission window. Images were recorded with 20 x and 40 x objectives. Cells of the control groups, immobilized on the glass bottom of the CELLVIEW dishes, were imaged similarly.

### Graphene-coating

Directly prior to STEM, the labelled cells on the microchips were covered with graphene sheets as described earlier (Dahmke et al. 2017). While viewing the sample using a binocular microscope, the graphene-NaCl crystal, held with tweezers, was slowly submerged under an angle of 20–45 ° with respect to the liquid surface in ~ 100 ml of HPLC-grade water in a clean glass beaker. Upon contact with water, the graphene sheet detached (within seconds) from the NaCl crystal, and floated on the water surface. The sheet was then carefully scooped up by submerging a microchip sample held by curved tweezers. The surface of the microchip was subsequently air-dried for a few minutes (Dahmke et al. 2017). During this process the graphene sheet sank onto the cells and the supporting SiN membrane, thus tightly wrapping and enclosing the cells so that the enclosed cells were maintained in liquid state.

### Liquid-phase STEM

The graphene-coated sample was imaged with dark field STEM (ARM 200, JEOL, Japan) to observe the individual QD-labelled HER2 positions. The used electron beam energy was 200 keV. Firstly, overview STEM images were acquired of the entire SiN window that served as orientation purpose. By comparing the STEM overview images to the fluorescence- and DIC images of the same sample, it was possible to relocate cells during electron microscopy, and to choose cells with sufficient HER2 labelling for the high-resolution STEM. High-resolution images of selected areas of cells were subsequently acquired for the measurement of the individual positions of QD-labels. The pixel dwell-time was 14 μs, and magnifications of 60,000–120,000 × were used, corresponding to pixel sizes between 1.66 and 0.66 nm. The image size was 2048 × 2048 pixels, yielding a scanning area of 7.0–2.8 μm^2^ per image, the recording time per image was approx. 1 min. The applied electron doses were in the range of 16–63 e^−^/Å^2^, well below the radiation damage limit of these samples (Dahmke et al. 2017).

### Statistical analysis

The label positions of QDs in STEM images were automatically detected using a plugin written in ImageJ (NIH) as described elsewhere (Peckys et al. 2015). In short, an image was first checked for artefacts such as larger debris particles, and some images were excluded for the analysis. When the local label density was too high (i.e. > 2.000 labels/μm^2^) for automatic detection, as was the case in a few membrane regions, data of these regions were omitted as well. A macro allowed automatic processing of a series of images, resulting in a data file with x/y-coordinates for each image. The measured label positions were then statistically analysed for all data files in a group using software of local design programmed in c++ in an Unix environment on OSX. The statistical analysis was based on calculating the pair correlation function *g*(*r*) (Stoyan and Stoyan 1996):

1 $$ g(r)=\frac{1}{\pi {p}^2 ry(r)}\sum \limits_{i=1}^N\sum \limits_{j=i+1}^Nk\left(r-\left|{x}_i-{x}_j\right|\right). $$

Here, *r* is the pair distance, *ρ* the labelling density, and *N* the number of labels. The covariance function (Stoyan et al. 1993) γ and the kernel (Fiksel 1988) *k* account for a correction at the edge of the image, and a smoothing of *g*(*r*), see also (Peckys et al. 2015). The centre-to-centre distance between the pair of labels at positions i and j is obtained from the modulus |**x**_i_ - **x**_j_| of the two-dimensional (x, y) position vector **x.** Pair distances smaller than 10 nm were excluded to avoid counting overlapping QDs, thus assuming all labels were located in one horizontal plane, which neglects curvature of the cell surface. The resulting *g(r)* histogram exhibited a bin width in *r* of 2.5 nm. The data of the images of one group were averaged by weighting the data of each image by its corresponding particle density. The time for a complete analysis of a series of selected images from a patient (images with too high label densities had to be excluded) was in the range of 20–30 min.

## Results

### Light microscopy of QD-labelled HER2 in tissue sections

**Fig. 1:**
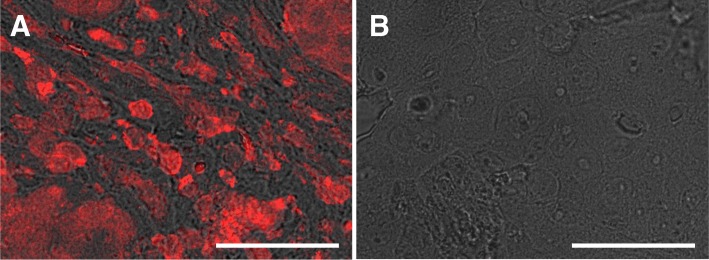
Light microscopy images demonstrating the specificity of HER2 affibody-QD labelling on tumour sections. The images represent an overlay of the fluorescence signal and the direct interference contrast (DIC) signal, providing a three-dimensional impression of the sample surfaces, and the red colour originating from the QD655’s fluorescence. **a** A tumour section exhibiting red fluorescence signals deriving from the HER2-bound QDs. The cell shapes are visible from the DIC signal in grey. **b** Example of a negative control experiment, performed with another section from the same tumour, and the same labelling protocol, except that the HER2 Affibody incubation was omitted. The exposure settings of the fluorescence channel were the same for both images. Scale bars 50 μm

Standard FFPE tissue blocks of HER2 positive and negative breast- and gastric cancer samples were studied at the single cell and single molecule level using light- and electron microscopy. All tumour samples were collected from the tissue archive of the Institute of Pathology of the University Medical Center Mannheim. The study was approved by the local ethics committee in Mannheim and was assigned with the number 2016-080R-MA. We first tested if it was possible to specifically label HER2 proteins in tissue material with fluorescent QD nanoparticles coupled via streptavidin to a biotin-Affibody compound specifically binding to HER2 in a 1:1 stoichiometry (Eigenbrot et al. 2010) as needed to examine HER2’s spatial distribution (Peckys et al. 2015). Three-micron thick tissue sections were prepared from FFPE blocks of HER2 positive and negative cancer samples (see Additional file 1 Figure S1). Two HER2 positive and one HER2 negative sample were selected for further microscopic analyses. After application of a two-step labelling protocol for HER2, as described earlier (Peckys et al. 2015), the tissue sections were examined with LM, using fluorescence contrast and DIC. A labelled tumour section is shown in Fig. 1a. Bright red fluorescence signals, emitted by the HER2-bound QD655 label, appeared on many cancer cells dispersed throughout the tissue section, and surrounded by non-fluorescent tissue, likely composed of fibroblasts and endothelial cells from blood vessel. Within the fluorescent cells, the signal was highest at the cell periphery, confirming that HER2 resided predominantly in the plasma membrane. Intracellular regions showed lower HER2 signals. Based on the known specificity of the anti-HER2-Affibody (Eigenbrot et al. 2010), it can thus be concluded that the protocol specifically labelled HER2 overexpressing cells but not the interspersed stromal or inflammatory cells. The specificity of the second labelling step was tested by applying the same protocol without the affibody-labelling step. The complete lack of red fluorescence signal in Fig. 1b proves that streptavidin-conjugated QDs bound solely to HER2-bound biotinylated affibodies. Non-specific binding of streptavidin-conjugated QDs is thus ruled out when incubated with tissue sections. The affibody-QD labelling protocol is possibly an alternative to test tumour tissue samples for HER2 expression with high specificity and high fluorescence brightness.

### Light microscopy of QD-labelled HER2 in dissociated cancer cells

**Fig. 2:**
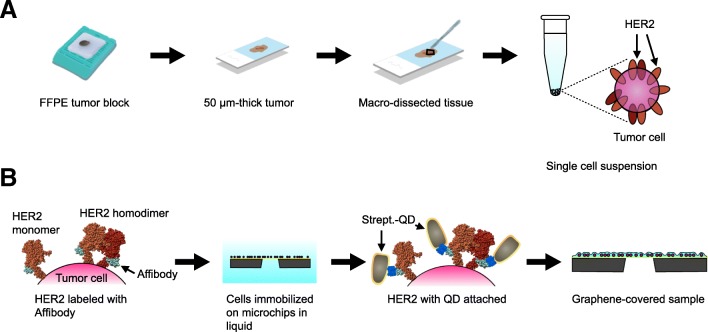
Schematic representation of the sample preparation method. **a** Retrieval of a single cell suspension from an FFPE tumour block. The tumour block is first sectioned. The tumour is then macro-dissected and transferred into a tube for single cell retrieval by collagenase and dispase digestion. **b** HER2 membrane proteins are specifically labelled with Affibody peptides. The cells are then immobilized on a microchip, and streptavidin Quantum Dot (Strept.-QD) is attached. The final step is the coverage of the sample with a graphene sheet

To study the spatial distribution of HER2 in the intact plasma membrane, tumour samples were dissociated into single cells, HER2 was labelled, and the samples were subsequently investigated with light and high-resolution electron microscopy. The dissociation protocol depicted in Fig. 2a yielded suspensions of dispersed, single cells. In short, the method involved preparation of 50-μm thick FFPE tissue sections, from which tumour regions were macro-dissected followed by deparaffinization and rehydration. To remove fixation-induced protein crosslinks, tumour tissue was then subjected to heat-induced antigen retrieval before enzymatic dissociation into single cells was performed. Membrane bound HER2 proteins on single cells were subsequently labelled with Affibody peptides (Fig. 2b). The protocol applied to the cell suspensions required centrifugation and resuspension steps until the affibody incubation was finished; this step was different from our original protocol (Peckys et al. 2015; Peckys and de Jonge 2015). Afterwards, the affibody-labelled cells designated for electron microscopic investigation (see below) were immobilized on silicon microchips with silicon nitride (SiN) windows (Ring et al. 2011). The microchip surfaces were previously covered with mussel adhesion protein to enhance cell adhesion. The immobilization of the cells facilitated and accelerated the handling during the second part of the labelling protocol, which included the chemical fixation and the incubation with QDs. Control samples for light microscopy were prepared in glass bottom dishes.

**Fig. 3:**
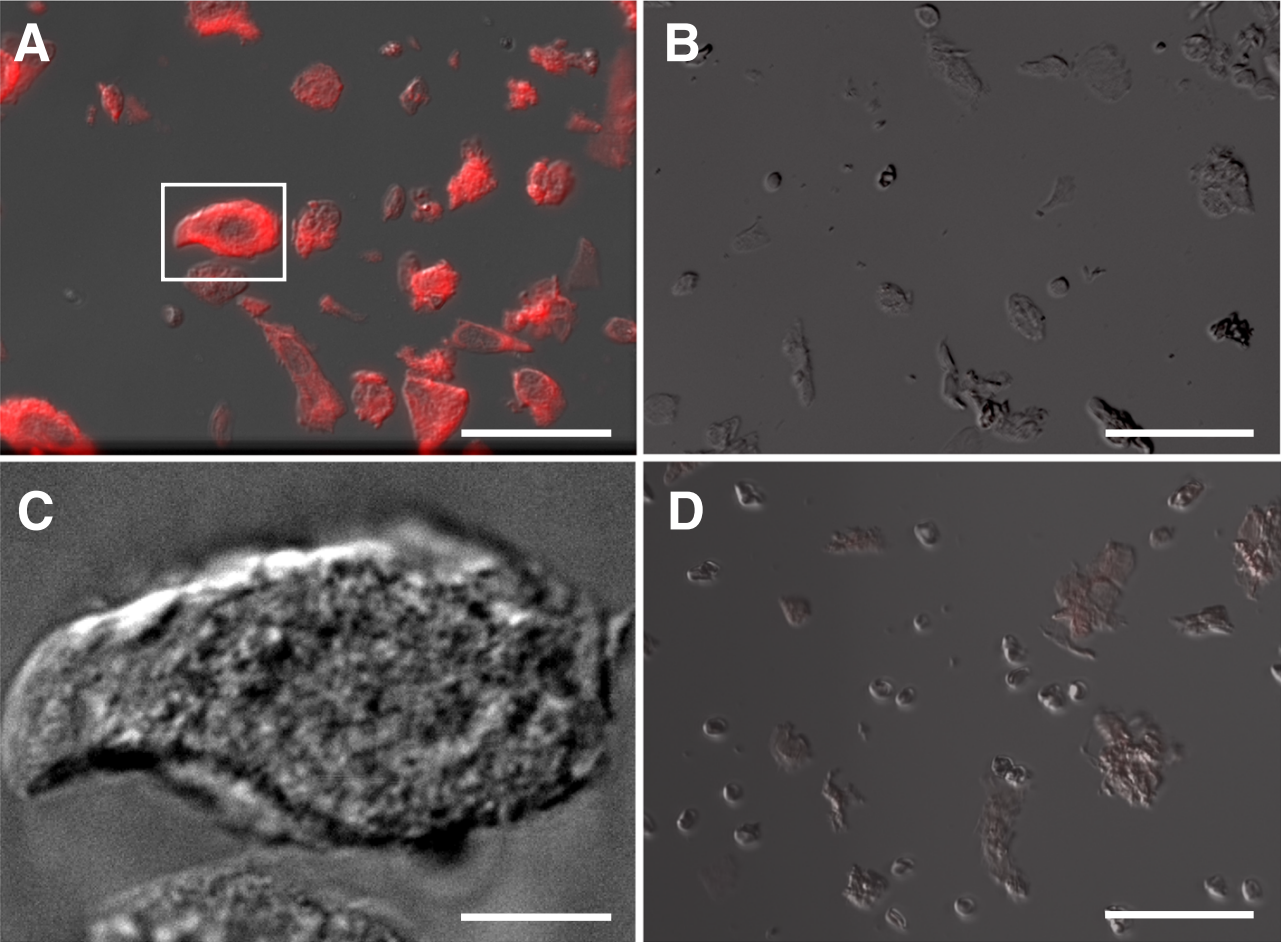
Light microscopy images demonstrating the specificity of HER2 affibody-QD labelling on dissociated tumour cells. **a** Dissociated tumour cells with labelled HER2. **b** Negative control without Affibody incubation for dissociated cells from the same sample as shown in **a**. **c** DIC image from the boxed cell in **a**, acquired with a 63x oil immersion objective. **d** Dissociated breast cancer cells classified as HER2 negative. The cells were immobilized on a microchip with a silicon nitride window in **a** and **c**. In **b**, and **d**, the sample was placed in a glass bottom cell culture dish. The images represent an overlay of the fluorescence- and DIC signals except in **c**. The exposure settings of the fluorescence channel were the same for images **a**, **b**, **d**, and **f**. Scale bars 50 μm in **a**, **b**, **d**; 10 μm in **c**

The HER2-labelled dissociated tumour cells were first studied with light microscopy. Figure 3a shows a combined DIC- and fluorescence-microscopy image of tumour cells with QD-labelled HER2, immobilized on the window area of a SiN membrane on a microchip. The tumour cells showed a fluorescence signal after HER2 labelling whereby the intensity varied between the individual cells, reflecting different levels of membrane-bound HER2. The control sample, prepared with the same protocol except that the affibody incubation was omitted, did not show a noticeable fluorescence signal (Fig. 3b). The labelling was thus HER2 specific also for this sample preparation protocol. In contrast to cell culture in which cells tend to flatten out when grown on a surface, the dissociated cells maintained a considerable thickness. As can be seen in Fig. 3c, depicting the marked cell in Fig. 3a with a higher resolution DIC image, this cell had a crinkled membrane surface and was not flattened at the cell borders. The same was observed for most other tumour cells. As a further control, we applied the HER2 labelling protocol also to a HER2 negative classified tumour sample. An exemplary overlay image of this control is shown in Fig. 3d. The fluorescence signal intensity is much smaller than for Fig. 3a showing that HER2 labelling of cells in a tumour classified as HER2 positive indeed leads to signal that is distinguishable from HER2 negatively classified samples, and thus verifies the specificity of the method. Only a dim, red shadow can be seen on some of the cells in Fig. 3d indicating the presence of HER2, albeit at a much lower level than in the HER2 positive cells (Bai et al. 2013).

### Liquid-phase STEM and quantitative analysis of QD-labelled HER2 in dissociated cancer cells from tumour tissues

**Fig. 4:**
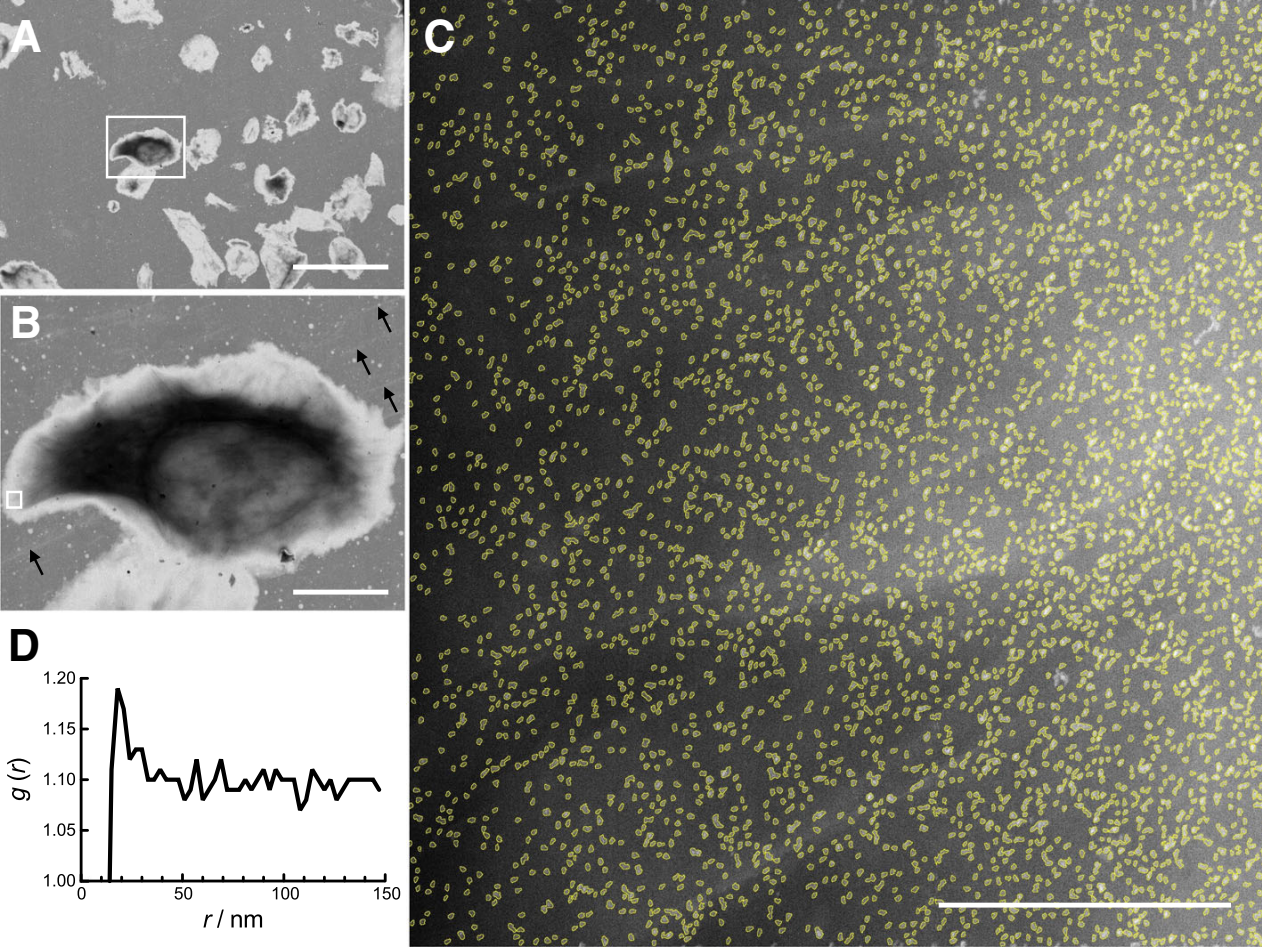
Scanning transmission electron microscopy (STEM) images of the same sample shown in Fig. 3a and c, after an additional fixation step with glutaraldehyde, and coating with multilayer graphene, keeping the cells in hydrated state inside the electron microscope. **a** Low magnification (800 x) overview STEM image of the same region as shown in Fig. 3c used to navigation to the regions of interest. **b** Low magnification (4000 x) overview STEM image of the boxed cell in **a**, the same as shown in Fig. 3c. The multilayer graphene sheet, covering the whole sample, was invisible, except for some faint, bright, linear structures, representing fine folds in the graphene (see arrows). **c** STEM image (120,000 x magnification) recorded at the boxed region in B revealing the abundance of labelled HER2 on the cancer cell. The electron-dense QD labels were automatically detected and outlined in yellow to enhance their visibility. **d** The pair correlation function *g*(*r*) measuring the probability of a certain inter-label distance *r* calculated for the labels shown in **c**. The peak at ~ 20 nm indicates the presence of HER2 homodimers. Scale bars, in **a**: 50 μm, in **b**: 10 μm, and in **c**: 500 nm

To examine the spatial distribution of HER2 proteins in the plasma membrane of a single tumour cell, samples prepared from dissociated cells were examined using liquid-phase STEM. Following LM imaging after QD attachment (Fig. 3), these samples were fixed with glutaraldehyde, and coated with multilayer graphene sheets (Fig. 2) to ensure the cells remained hydrated in the high vacuum of the specimen chamber of the electron microscope (Dahmke et al. 2017; Textor and de Jonge 2018). STEM started by recording low magnification (800–1000 x) overview images, serving orientation and easy navigation to the cellular regions of interest. The cells that attach on the window area of a microchip represent a small random selection of the dissociated tumor cells. From these cells, a number of HER2 positive cells, representing the detected range of HER2 expression as previously detected with light microscopy, were selected and examined with STEM. Figure 4a shows a STEM overview image of the same microchip window area shown in Fig. 3a. At higher magnification (Fig. 3b), the marked area displays an irregular, slightly frayed cell periphery and the nuclear region can be discerned. The STEM contrast results in a much different image than the DIC contrast of Fig. 3c. At the left border, a small square indicates the position of the recorded high-resolution STEM image shown in Fig. 4c. Here, the abundantly present QD-labelled HER2 molecules on the plasma membrane are visible. The QDs have an elongated shape, with an electron dense core of ~ 7 × 13 nm^2^, generating a bright contrast when imaged in dark field STEM mode. The automatic detection software outlines all detected QDs (in yellow). In this image, covering an area of 2.89 μm^2^, 3890 particles were detected, corresponding to 1350 detected HER2/μm^2^. The projected two-dimensional area of the surface covered by this cell is ~ 730 μm^2^, this would yield a total of about one million HER2 molecules for this cell. However, this value has to be multiplied at least by a factor of 2, because approximately the same amount of membrane surface covered the other side of the cell, positioned close to the SiN membrane. The estimated value of ~ 2 million HER2 receptors per cell lies well in the range of HER2 overexpressing cancer cell lines used for basic research (Onsum et al. 2013).

Because the software also determines the lateral (x, y) position of the centre of each label, these collected data can be used for further statistical analysis via the pair correlation function (Peckys et al. 2015; Stoyan and Stoyan 1996). This was done for the detected particles found in the image in Fig. 4c, and the resulting pair correlation function *g*(*r*) is shown as function of the centre-to-centre label distance *r* in Fig. 4d. *G*(*r*) values > 1 prove a higher incidence of the corresponding spatial frequency than a random distribution. Since the *g*(*r*) for Fig. 3c exhibits a clear peak at ~ 20 nm, an inter-label distance of 20 nm thus appeared with a higher likelihood than random occurrence, indicating the presence of HER2 homodimers, as was also found in HER2 overexpressing SKBR3 cells (Peckys et al. 2015; Peckys et al. 2017). The *g*(*r*) also shows that pair distances beyond the peak value do not return to 1 but remain higher, which presumably reflects the curved topography of the cell surface.

**Fig. 5:**
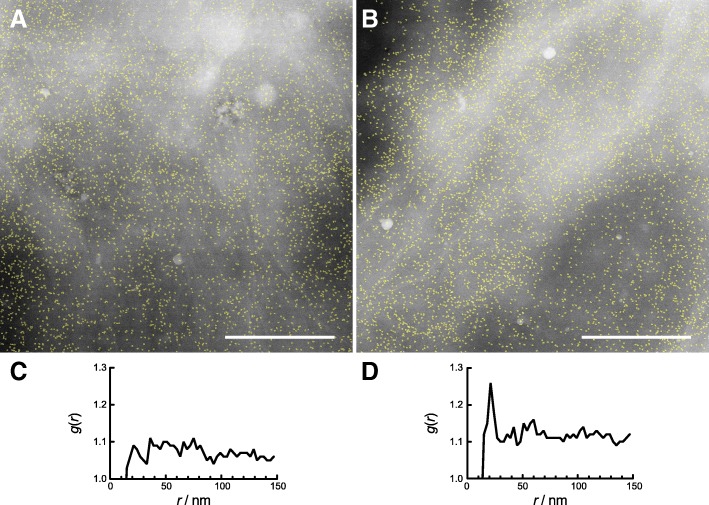
High magnification STEM images (80,000 x) from neighbouring regions on the same cell differing in their content of HER2 homodimers. **a** The image shows a total of 4362 detected particles with a density of 823 particles/μm^2^. **b** A similar image, recorded only a few microns away from the first image, and with a similar number of labels and label density value (3567 detected particles and 680 particles/μm^2^). **c** The *g*(*r*) analysis corresponding to A has no peak and thus indicates a lack of HER2 homodimers in this membrane region. **d** The *g*(*r*) for image **b** reveals a 20-nm peak indicating the presence of HER2 homodimers. Scale bars in **a** and **b**: 500 nm

**Fig. 6:**
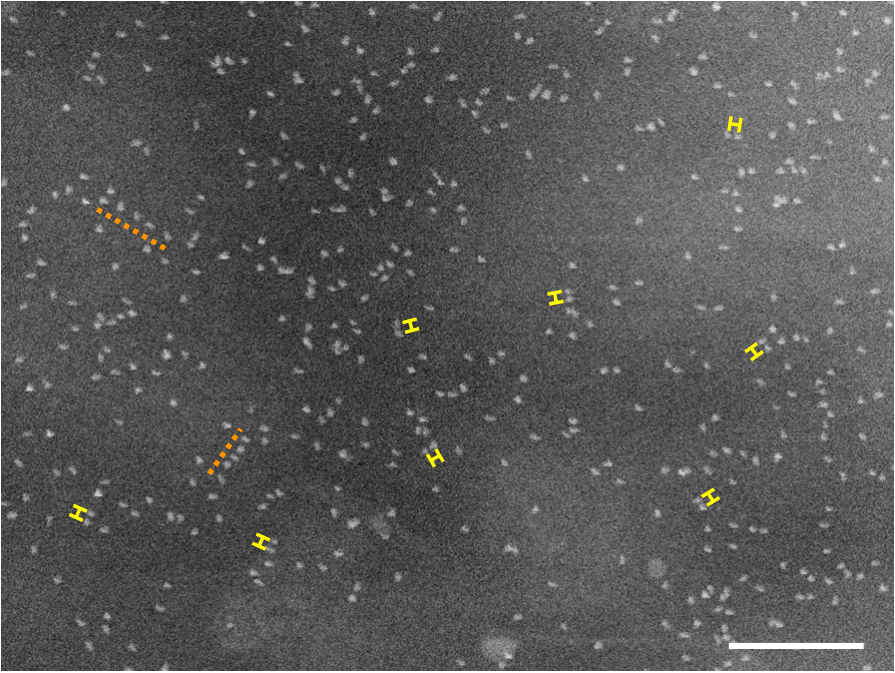
STEM image (80,000 x) from a cell with a label density of 413 particles/μm^2^ sufficiently low to allow the recognition of typical spatial distribution features of overexpressed HER2 by eye. Examples of label pairs, presumably, HER2 homodimers, are indicated by yellow, 20 nm long rulers. Two examples of occasionally occurring, linear arrangements of HER2 are indicated by the orange, dotted lines. Scale bar: 200 nm

Cells from two different patients were examined with STEM yielding data from 8, respectively 5 cells. From each cell, 5 to 12 images were recorded, 52 images were analyzed from patient 1, and 30 images from patient 2, two examples are show in Fig. 5. A typical example for a membrane region without HER2 homodimer peak is shown in Fig. 5a, with the corresponding *g*(*r*) in Fig. 5c, and for a region with evidence for HER2 homodimers in Fig. 5b, and the *g*(*r*) depicted in Fig. 5d. It is not possible to discern the difference between cell regions with- and without HER2 homodimers by eye, especially while the average label densities are as high as 832 and 680 labels/μm^2^ in Fig. 5a and b, respectively. Visual inspection of other images with lower label densities revealed several pairs of labels indicating HER2 homodimers, and also some peculiar, linear receptor arrangements (Fig. 6). The features of linear arrangements were previously found in HER2 overexpressing SKBR3 and HCC1954 cell lines (Parker et al. 2018). It was also possible to view the QD labels using transmission electron microscopy (TEM) instead of STEM (Additional file 1 Figure S2). The contrast obtained in TEM is lower than in STEM and inverse. But since TEM is more commonly used in biological- and pathology laboratories, this example was added to show that the technique is broadly applicable.

The microscopy data acquired of both patients contained total membrane areas of 532 and 173 μm^2^ of which lateral position data of 106.828 and 119.201 detected labels were determined for patient 1 and 2, respectively. The respective areal densities of membrane bound HER2 were thus at least 201 and 689 proteins/μm^2^, and may even be larger because the labelling efficiency presumably did not reach 100%. Each image was automatically processed to gain data on the detected number of labels, their density and the corresponding *g*(*r*). The peak of the *g*(*r*) curve at 20 nm associated with the presence of HER2 homodimers was found in 32 and 55% of the imaged areas for HER2 positive patient 1, respectively HER2 positive patient 2. The lack of homodimers in some cell membrane areas is consistent with earlier data from SKBR3 cells were HER2 homodimers appeared predominantly on membrane ruffles (Peckys et al. 2015). However, it was impossible to discern such flat and ruffled regions on the examined patient tumour cells. The tumour cell areas with HER2 homodimers exhibited a 61 and 22% higher label density than the areas without homodimers for patient 1 and 2, which is consistent with earlier data on SKBR3 cells (Peckys et al. 2015).

## Discussion

HER2 overexpression is a major oncogenic driver, and has been reported as relevant in many tumour types (Yan et al. 2015). For breast and gastric cancer, successful clinical studies have also proven effectiveness for anti-HER2 targeted drugs, and are now implemented as standard of care treatment (Piccart-Gebhart et al. 2005; Bang et al. 2010). The prerequisite for the prescription of these drugs is the presence of a high amount of HER2 molecules either on the tumour surface or by increased HER2 gene copies. While HER2 presence in general can be examined by immunohistochemistry on the protein level or with fluorescence in-situ hybridization on the DNA level, at least one major problem impairs HER2 testing. By measuring the amount of HER2 molecules no assessment of HER2 activation is done. Not the amount of HER2 molecules, which is measured indirectly by immunohistochemistry, is responsible for the malignant transformation of the tumour cells, but rather the activation of the HER2 pathway. Since estimation of HER2 homodimerization is technically difficult, conventional immunohistochemical methods so far, evaluate the amount of HER2 molecules as a proxy, although knowing that this is only the first step in the HER2 activating cascade. It has been postulated that the excessive presence of HER2 molecules also leads to the formation of HER2 homodimers, which in turn activate the HER2 signalling pathway. But as we have shown earlier at the single-cell level, the overexpression of HER2 does not always lead to the formation of HER2 homodimers in rare subpopulations of resting, or quiescent cancer cells (Peckys et al. 2015; Peckys et al. 2017). This might possibly play a role in the context of primary resistance to Trastuzumab, as found for instance in ~ 20% of early stage breast cancer patients, and ~ 70% of patients with metastatic disease (Wilken and Maihle 2010).

Considering the impact of any information about the HER2 status for cancer diagnosis and therapy, the relative scarcity of studies concerning HER2 homodimers is striking. Of all ~ 37,500 publications appearing for the search terms “HER2” or “ErbB2” in the Web of Science Collection, covering the time span from 1987 till now, less than 0.2% included the search term “homodimer”. This protein complex seems to be notoriously difficult to study. Biochemical methods can in principle be used to detect homodimers in pooled cellular material. But this technique is unreliable for HER2 because its extracellular domain does not homodimerize in solution (Arkhipov et al. 2013; Badache and Hynes 2004). Moreover, contradicting structural models have been published (Arkhipov et al. 2013; Hu et al. 2015). Light microscopy based techniques do not resolve homodimers from monomers due to lack of spatial resolution and the high surface density of endogenous HER2 in the plasma membrane or other limitations as discussed elsewhere (Peckys et al. 2015; Zehbe et al. 2010). Although the abundance of HER2 can be measured via a range of methods, it is thus impossible to reliably detect the presence of the HER2 homodimers. For example, one existing method that presumably detects the presence of homo- and heterodimers is the so-called proximity ligation assay (PLA) (Soderberg et al. 2006). In PLA, a fluorescence signal is generated when two complementary antibody-labelled molecules each attached to a HER2 protein are in close proximity. However it is known that “..highly expressed proteins should have an increased risk of false positive PLA signal production as more copies will reside close to the interaction partner by chance” (Leuchowius et al. 2013). The separation distance between two molecules recognized by PLA depends on the size of the antibodies (~ 15 nm in size) plus the attached oligonucleotide chains, and reaches several tens (Leuchowius et al. 2013) and possibly 40 nm (Aslemarz et al. 2018). As we show in our data, the surface density of HER2 readily reaches a value of 700 proteins/μm^2^ for typical HER2+ tumour samples, so that 3–4 proteins would be within an area of radius 40 nm. A positive PLA signal would thus be obtained regardless whether dimers are present. Another complicating factor is a non-linear dependence of the PLA with protein density (Mocanu et al. 2011) possibly explained by steric hindrance of the antibody binding (Leuchowius et al. 2013), indicating that the binding of two antibodies, necessary for a PLA signal, to a real dimer might not even occur due to the small size (~ 7 nm) of the dimer. At clinically relevant HER2 densities, PLA thus yields false positive and seemingly specific signals, even for totally unrelated antigens (Aslemarz et al. 2018).

The above results demonstrate the feasibility of a single-molecule based evaluation of HER2 homodimerization on patient tumour cells, classified by IHC as HER2 3+. The combination of specific HER2 labelling via an affibody peptide (Peckys et al. 2015), and high resolution liquid phase STEM of graphene coated intact cells (Dahmke et al. 2017), allowed the analysis of hundred thousands of individual HER2 positions for endogenous protein expression levels. Automated image analysis tools, including the pair correlation function, revealed the presence of HER2 homodimers on a fraction of the examined cell membrane regions. A key feature of the presented method is the capability to conduct the analysis on a cell-to-cell basis such that differences between tumour cells are readily observed and the topic of cancer cell heterogeneity is addressed. We anticipate that it will be possible to develop labels for HER1, and HER3 as well, so that both homodimers and heterodimers involving all HER1-HER3 can be analysed in future.

The correlative light- and electron microscopy method described here, could take pathological analysis a step further by examining HER2 homodimerization directly for patients’ biopsy samples. In further projects we will have to evaluate if the latter examination surpasses conventional HER2 testing with respect to a predictive value, and helps to identify anti-HER2 responder in a more sufficient way (Wilken and Maihle 2010). The singe-molecule based information about HER2’s spatial distribution and homodimerization level will possibly provide clinicians a new quantitative guidance for a personalized antibody-based drug therapy. In addition to providing unique information about the levels of HER2 homodimers, it also provides accurate measures of the areal density of HER2 per cell and locally within a cell. This measure may possibly support standard methods used in the pathology laboratories to classify HER2 positive tumour, which have to be interpreted with care, since, for example, discordant results are reported between genomic and transcriptomic analyses (Allred et al. 2012; Ross and Fletcher 1998).

Besides gaining quantitative information about HER2 homodimers, it would also be important to have such data from HER2 heterodimers, which have been attributed pivotal roles in HER2 oncogenic function, as their presence is expected to be highly relevant for targeted therapy (Holbro et al. 2003; Qian et al. 1994; Roskoski 2014). Development of multiplexed labelling and analysis methods for two other important members of the EGFR family, mainly EGFR (or HER1) and HER3, is feasible by using for instance a combination of QDs emitting at 655 nm, having a large electron dense core of a bullet-type shape (used in this study), and QDs emitting at 565 nm, with a much smaller core size, and a round shape.

## Conclusions

These results show that is possible to measure the locations of individual HER2 proteins in dissociated, HER2-overexpressing tumour cells from FFPE tumour tissue. The position information is readily used to measure the HER2 areal density, to probe for the presence of HER2 homodimers, and to visualize other characteristic features of spatial correlations, such as linear arrangements. It was thus found that the areal density of HER2 in the plasma membranes of patient’s cells were on average 201, respectively 689 proteins/μm^2^. Cell signalling active HER2 homodimers were found in 32 and 55% of the acquired images of patient 1 and 2, respectively. The described analytical method could take HER2 testing a step further by examining HER2 homodimerization directly out of FFPE tumour tissue and therefore allows a deeper and more precise characterization of the patients’ HER2 status. Such new information will help to improve conventional HER2 classification in terms of areal density of HER2 in the plasma membrane, the amount of cell growth signalling active HER2 homodimers, and a quantification of differences between cells.

## Additional file

Additional file 1: **Figure S1.** representative images of histology and HER2 immunohistochemistry. **Figure S2.** exemplary TEM images recorded from the sample 1. (PDF 8490 kb)

## Data Availability

Please contact the authors for data requests.

## Abbreviations

BSA: Bovine Serum albumin Fraction V
CB: Cacodylate buffer
DIC: Direct interference contrast
FFPE: Formalin-fixed paraffin-embedded
GA: Glutaraldehyde
GLY: Glycine
HPLC: High pressure liquid chromatography
IHC: Immunohistochemistry
LM: Light microscopy
PBS: Phosphate buffered saline
PLA: Proximity ligation assay
PLL: Poly-L-lysine
PMMA: Poly-methyl-methacrylate
QD: Quantum dot
S: Saccharose
SiN: Silicon nitride
STEM: Scanning transmission electron microscopy
TEM: Transmission electron microscopy

## References

[CR1] Allred DC, Bhargava R, Dabbs D, Davide J, Dabbs DJ (2012). Predictive and prognostic marker testing in breast pathology: immunophenotypic subclasses of disease. Breast Pathol.

[CR2] Arkhipov A, Shan YB, Kim ET, Dror RO, Shaw DE (2013). Her2 activation mechanism reflects evolutionary preservation of asymmetric ectodomain dimers in the human EGFR family. Elife.

[CR3] Aslemarz A, Lasko P, Fagotto F. Limited significance of the in situ proximity ligation assay. *bioRxiv*. 2018:411355. 10.1101/411355.

[CR4] Badache A, Hynes NE (2004). A new therapeutic antibody masks ErbB2 to its partners. Cancer Cell.

[CR5] Bai Y, Cheng H, Bordeaux J (2013). Comparison of HER2 and phospho-HER2 expression between biopsy and resected breast cancer specimens using a quantitative assessment method. PLoS One.

[CR6] Bang YJ, Van Cutsem E, Feyereislova A (2010). Trastuzumab in combination with chemotherapy versus chemotherapy alone for treatment of HER2-positive advanced gastric or gastro-oesophageal junction cancer (ToGA): a phase 3, open-label, randomised controlled trial. Lancet.

[CR7] Beuzeboc P, Pouillart P, Scholl S (2001). Targeting HER2 in other tumor types. Ann Oncol.

[CR8] Bolognesi C, Forcato C, Buson G (2016). Digital sorting of pure cell populations enables unambiguous genetic analysis of heterogeneous formalin-fixed paraffin-embedded tumors by next generation sequencing. Sci Rep.

[CR9] Dahmke IN, Verch A, Hermannsdorfer J (2017). Graphene liquid enclosure for single-molecule analysis of membrane proteins in whole cells using Electron microscopy. ACS Nano.

[CR10] Di Fiore P, Pierce J, Kraus M, Segatto O, King C, Aaronson S (1987). erbB-2 is a potent oncogene when overexpressed in NIH/3T3 cells. Science.

[CR11] Eigenbrot C, Ultsch M, Dubnovitsky A, Abrahmsen L, Hard T (2010). Structural basis for high-affinity HER2 receptor binding by an engineered protein. Proc Natl Acad Sci.

[CR12] Fiksel T (1988). Edge-corrected density estimators for points processes. Statistics.

[CR13] Holbro T, Beerli RR, Maurer F, Koziczak M, Barbas CF, Hynes NE (2003). The ErbB2/ErbB3 heterodimer functions as an oncogenic unit: ErbB2 requires ErbB3 to drive breast tumor cell proliferation. Proc Natl Acad Sci.

[CR14] Hu S, Sun Y, Meng Y (2015). Molecular architecture of the ErbB2 extracellular domain homodimer. Oncotarget.

[CR15] Hudziak RM, Schlessinger J, Ullrich A (1987). Increased expression of the putative growth factor receptor p185HER2 causes transformation and tumorigenesis of NIH 3T3 cells. Proc Natl Acad Sci.

[CR16] Leuchowius KJ, Clausson CM, Grannas K (2013). Parallel visualization of multiple protein complexes in individual cells in tumor tissue. Mol Cell Proteomics.

[CR17] Lonardo F, Di Marco E, King CR (1990). The normal erbB-2 product is an atypical receptor-like tyrosine kinase with constitutive activity in the absence of ligand. New Biol.

[CR18] Menard S, Casalini P, Campiglio M, Pupa S, Agresti R, Tagliabue E (2001). HER2 overexpression in various tumor types, focussing on its relationship to the development of invasive breast cancer. Ann Oncol.

[CR19] Mocanu M-M, Váradi T, Szöllősi J, Nagy P (2011). Comparative analysis of fluorescence resonance energy transfer (FRET) and proximity ligation assay (PLA). Proteomics.

[CR20] Onsum MD, Geretti E, Paragas V (2013). Single-cell quantitative HER2 measurement identifies heterogeneity and distinct subgroups within traditionally defined HER2-positive patients. Am J Pathol.

[CR21] Parker K, Trampert P, Tinnemann V, Peckys D, Dahmen T, de Jonge N (2018). Linear chains of HER2 receptors found in the plasma membrane using liquid-phase Electron microscopy. Biophys J.

[CR22] Peckys DB, de Jonge N (2015). Studying the stoichiometry of epidermal growth factor receptor in intact cells using correlative microscopy. J Vis Exp.

[CR23] Peckys DB, Korf U, de Jonge N (2015). Local variations of HER2 dimerization in breast cancer cells discovered by correlative fluorescence and liquid electron microscopy. Sci Adv.

[CR24] Peckys DB, Korf U, Wiemann S, de Jonge N (2017). Liquid-phase electron microscopy of molecular drug response in breast cancer cells reveals irresponsive cell subpopulations related to lack of HER2 homodimers. Mol Biol Cell.

[CR25] Piccart-Gebhart MJ, Procter M, Leyland-Jones B (2005). Trastuzumab after adjuvant chemotherapy in HER2-positive breast cancer. N Engl J Med.

[CR26] Qian XL, Levea CM, Freeman JK, Dougall WC, Greene MI (1994). Heterodimerization of epidermal growth-factor receptor and wild-type or kinase-deficient Neu - a mechanism of Interreceptor kinase activation and transphosphorylation. Proc Natl Acad Sci.

[CR27] Ring EA, Peckys DB, Dukes MJ, Baudoin JP, de Jonge N (2011). Silicon nitride windows for electron microscopy of whole cells. J Microsc.

[CR28] Roskoski R (2014). The ErbB/HER family of protein-tyrosine kinases and cancer. Pharmacol Res.

[CR29] Ross JS, Fletcher JA (1998). The HER-2/neu oncogene in breast cancer: prognostic factor, predictive factor, and target for therapy. Stem Cells.

[CR30] Soderberg O, Gullberg M, Jarvius M (2006). Direct observation of individual endogenous protein complexes in situ by proximity ligation. Nat Methods.

[CR31] Stoyan D, Bertram U, Wendrock H (1993). Estimation variances for estimators of product densities and pair correlation functions of planar points processes. Ann Inst Stat Math.

[CR32] Stoyan D, Stoyan H (1996). Estimating pair correlation functions of planar cluster processes. Biom J.

[CR33] Textor Martin, de Jonge Niels (2018). Strategies for Preparing Graphene Liquid Cells for Transmission Electron Microscopy. Nano Letters.

[CR34] Weatherup RS, Shahani AJ, Wang ZJ, et al. In situ graphene growth dynamics on polycrystalline catalyst foils. Nano Lett. 2016. 10.1021/acs.nanolett.6b02459 PMC5064306 27576749

[CR35] Wilken JA, Maihle NJ (2010). Primary trastuzumab resistance: new tricks for an old drug. Ann N Y Acad Sci.

[CR36] Wolff AC, Hammond MEH, Allison KH (2018). Human epidermal growth factor receptor 2 testing in breast Cancer: American Society of Clinical Oncology/College of American Pathologists Clinical Practice Guideline Focused Update. J Clin Oncol.

[CR37] Yan M, Schwaederle M, Arguello D, Millis SZ, Gatalica Z, Kurzrock R (2015). HER2 expression status in diverse cancers: review of results from 37,992 patients. Cancer Metastasis Rev.

[CR38] Yarden Y, Sliwkowski MX (2001). Untangling the ErbB signalling network. Nat Rev Mol Cell Biol.

[CR39] Zehbe R, Haibel A, Riesemeier H (2010). Going beyond histology. Synchrotron micro-computed tomography as a methodology for biological tissue characterization: from tissue morphology to individual cells. J R Soc Interface.

