# Supplementary material for: Visualisation of HER2 homodimers in single cells from HER2 overexpressing primary formalin fixed paraffin embedded tumour tissue
Source: Mol Med. 2019 Aug 28;25:42. doi: 10.1186/s10020-019-0108-z (PMC6712713; doi:10.1186/s10020-019-0108-z)
Supplement: Supplementary file 1 — Figure S1. representative images of histology and HER2 immunohistochemistry. Figure S2. exemplary TEM images recorded from the sample 1. (PDF 8490 kb) [file 10020_2019_108_MOESM1_ESM.pdf]

Supplementary Information

Visualisation of HER2 homodimers in single cells from HER2 overexpressing primary formalin fixed paraffin embedded tumour tissue

Diana B. Peckys, Daniela Hirsch, Timo Gaiser, and Niels de Jonge

Figures S1, and S2

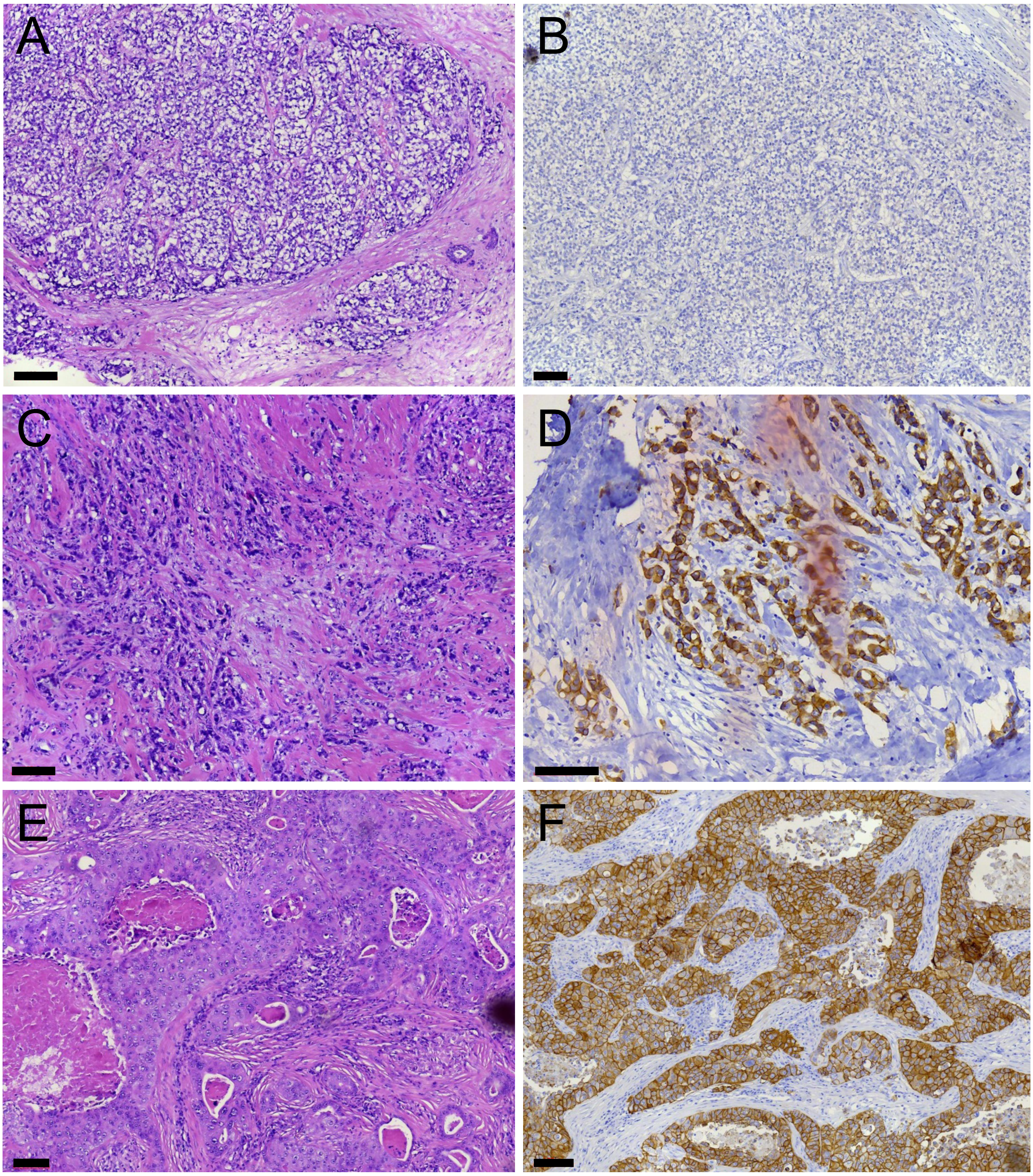

Figure S1. Representative images of histology (A, C, E) and HER2 immunohistochemistry (B, D, F) of the three tumour samples used in this study. (A, B) HER2 negative (Dako Score 0) breast cancer sample, (C, D) HER2 positive (Dako Score 3+) gastric cancer sample (patient 1) and (E, F) HER2 positive (Dako Score 3+) breast cancer sample (patient 2). While membranous staining of HER2 is absent in the HER2 negative case, the two HER2 positive cases show a very strong, circular HER2 staining of tumour cells. Scale bars: 100 µm

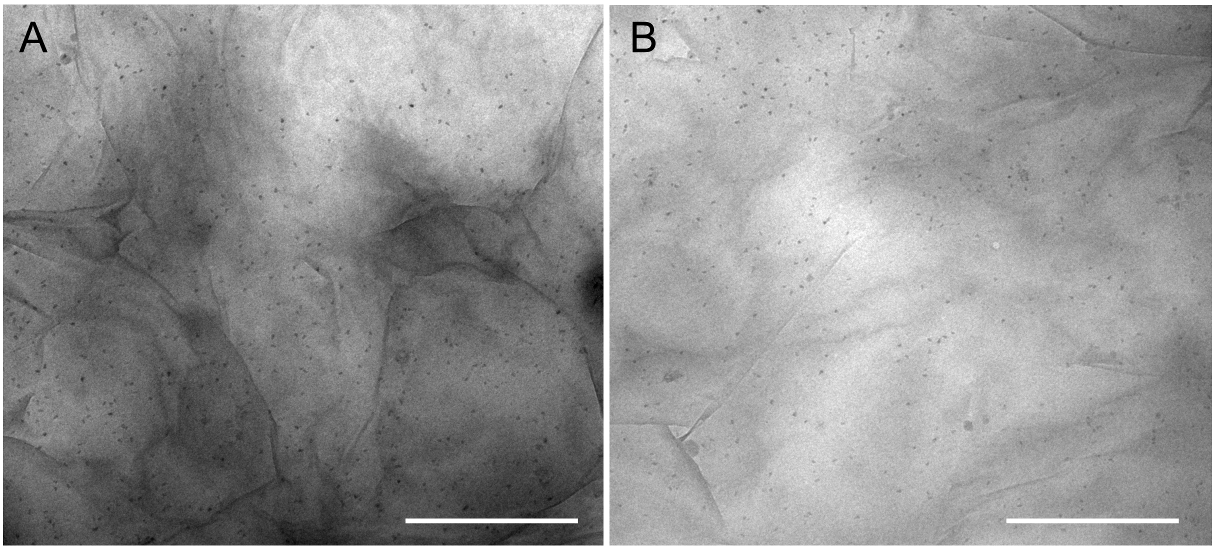

Figure S2. Two exemplary high-resolution (60,000 x magnification) transmission electron microscopy (TEM) images recorded from the same sample of patient 1. (A, B) These images show that quantum dot labelled HER2 on graphene-covered, hydrated cells of several microns thickness can also be visualized by TEM. The pixel size of this image was 1.13 nm, acquisition time was 5 s. Scale bars 500 nm.
